# Famous Poisoners in Fiction

**Published:** 1893-08-12

**Authors:** 


### Famous Poisoners in Fiction.

### II.—A DAUGHTER OF DARKNESS.

In the preface to the edition of 1853 Lord Lytton writes of "The Caxtons, a Family Picture," and "Lucretia; or, the Children of Night," that "the two fictions are intended as pendants, both serving, amongst other collateral aims and objects, to show the influence of home education—of early circumstances and example—on character and conduct." It is for this reason then that in this marvellously powerful, if awful, novel we have the veil removed from the life of the heroine, Lucretia Clavering, in a series, as it were, of dissolving-view glimpses.

First behold the girl, full of hope, of plans, of "love's delicious madness," of proud self-will; next, the woman, heartbroken, thwarted, deceived by him she loved; then face to face with death—death in its most awful form, and with her only chance of escape the offering up on the altar of crime a substitute; next, a woman, stern and haughty by nature, revenging herself passionately on the man she once loved, and proving by her cruel persistency that indeed

> Heaven has no rage like love to hatred turned,
> Nor hell a fury like a woman scorned.

Then, in the chapter called "Retrospect" is described the character of the second husband of Lucretia, the man she marries because he announces himself to be good, but whom she discovers to be in reality avaricious, mean, proud, and so hypocritical as to pretend to possess untold wealth to win her small dowry so as to save himself by means of her money from bankruptcy. In anger she leaves him; he follows her, *strikes* her. Incensed, scornful, the possessor of a deadly secret, this tempted, maddened, child of night then returns to him with the awful determination to rid herself of him most effectually.

Another scene. The mother's heart is wrung with a passionate longing to find again her lost son, he in whom her love and her ambition is centred, and whom she believes she has discovered in a noble, grand, strong-souled man, ambitious, proud, and destined assuredly to succeed in life's long struggle.

In the last scene the murderess mother, beholding in her son her victim, is driven by the horror of that terrible moment into insanity, "In that awful hour reason fled," we read, "till the Judgment Day from the blackened ruins of her lost soul." As an analyser of character, this master novelist, this great student in the science of physiology is almost unsurpassed, and never had he finer subject than in this strange, awful, yet grand woman, grand in her mighty wickedness; this woman whose force of will, fiery energy, great determination, firm perseverance, and brilliant intellectual powers arouses a strange admiration, even whilst the reader revolts with horror at her deeds of darkness.

> Oh, saddest words of tongue and pen
> Are those four words "It might have been."

How differently might not her character have developed had love not failed her and left her to the terrible guidance of those twin giants of evil, Ambition and Mammon, or if she had not had, to quote Lord Lytton's own words, "the fiend for her familiar."

This quotation recalls to mind another poisoner described in this book even Dalibard, the evil genius of the novel. The evil that he did, indeed, lived after him in the works of Lucretia and Varney. Dalibard is an ideal poisoner; he loves the art for its own sake; he loves the subtle power it gives to him. To quote his own words, "It is a mighty thing to feel in one's self that one is an army—more than an army. What thousands, aye, millions of men with trumpet and banner, and under the sanction of glory, strive to do—*destroy a foe*, that, with little more than an effort of the will, with a drop, a grain, for all his arsenal, one man can do." He loves the deed itself, not only its consequences. This same feeling is inherited by his beautiful son with "the cruel heart." The novelist rightly insists so particularly on the sweetness of the countenance of this vile villain, the stamp of crime rests only on the brow of the brutal sinner, and there is no brutality in the administering of the deadly potion; poisoning is, emphatically, *murder as a fine art*. And as we all know, to our cost, not only in Denmark and in the sixteenth century

> A man may smile and smile and be a villain.

Yet cruel, vicious, terribly, bitterly sarcastic as is Gabriel Varney, a feeling of pity for him arises as we read one remark put into his lips—

> "The father slew the mother," answered Gabriel.

His punishment is also perfect in its subtle horror; he who loved art, beauty, comfort, but too well, chained to that human bogey the bodysnatcher. But though the able novelist has drawn for us with intense power a judgment worthy of the sinner, yet the ending of the first edition is the truest to nature, if not to the voice of "poetic justice," for crime does usually succeed—obtain its ends—win its goal.

> The wicked flourish as a green bay tree.

The poison given by Lucretia to her strangely-saved victim seems to have been the Acqua di Tophana, a colourless liquidwithout any odour. To the sight this poison is pure as purest water, although the instrument of death to him who unwittingly quaffs the delicious, apparently wholesome, draught. It is a slow, mysterious poison; its symptoms being those of a nervous fever.

Lucretia and Gabriel are no fanciful characters; they are drawn from life, and the means employed (even that which seems most far-fetched, the poisoned ring) "have their foundations in literal facts." "Nor have I," the author writes, "much altered the social position of the criminals, nor in the least overrated their attainments and intelligence. In those more salient essentials which will, perhaps, most provoke the reader's incredulous wonder I narrate a history, not invent a fiction."

Alas! crime, even in these more civilised times still walks a gaunt spectral figure to and fro upon the earth. The art of poisoning is not yet one of the fine arts of the past. Still "the children of the night" haunt our paths and are "about our beds"; their shadow yet falls across our way through life. Still in the great cities that hydra-headed octopus, the poisoned cup, clasps in its deadly coils its unhappy victims, and deeds are wrought which the joyous Helens, the happy, boyish Percivals of to-day, in their purity, their innocence, "ne'er dream of, ne'er wot of." It is well, may be, that half the world does not know how the other half lives, for alas! "sorrow with knowledge entereth, and the wiser man is usually also a sadder one" (" Lucretia, or the Children of Night," by the Right Hon. Lord Lytton.)

